# Sustainability and dynamics of outcrop-to-outcrop hydrothermal circulation

**DOI:** 10.1038/ncomms8567

**Published:** 2015-06-26

**Authors:** Dustin M. Winslow, Andrew T. Fisher

**Affiliations:** 1 Earth and Planetary Sciences Department, University of California Santa Cruz, Santa Cruz, California 95064, USA; 2 Institute for Geophysics and Planetary Physics, University of California Santa Cruz, Santa Cruz, California 95064, USA

## Abstract

Most seafloor hydrothermal circulation occurs far from the magmatic influence of mid-ocean ridges, driving large flows of water, heat and solutes through volcanic rock outcrops on ridge flanks. Here we create three-dimensional simulations of ridge–flank hydrothermal circulation, flowing between and through seamounts, to determine what controls hydrogeological sustainability, flow rate and preferred flow direction in these systems. We find that sustaining flow between outcrops that penetrate less-permeable sediment depends on a contrast in transmittance (the product of outcrop permeability and the area of outcrop exposure) between recharging and discharging sites, with discharge favoured through less-transmissive outcrops. Many simulations include local discharge through outcrops at the recharge end of an outcrop-to-outcrop system. Both of these characteristics are observed in the field. In addition, smaller discharging outcrops sustain higher flow rates than larger outcrops, which may help to explain how so much lithospheric heat is extracted globally by this process.

## Abstract (web summary)

Much hydrothermal circulation occurs away from the mid-ocean ridges and out on ridge flanks, affecting lithospheric heat deficit, solute fluxes, and influencing the biosphere. Here, the authors use 3D simulations to look at what controls the circulation and flow rate between and through seamounts.

## Graphical abstract

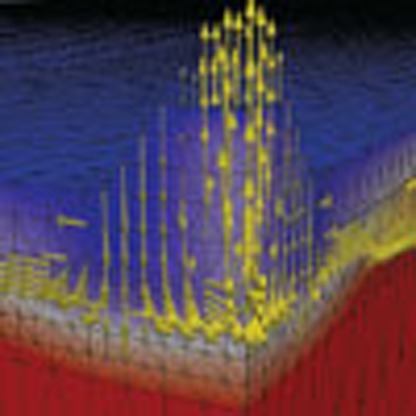

Ridge–flank hydrothermal circulation through the volcanic ocean crust is responsible for the majority of the seafloor heat-flux deficit^1^, drives solute fluxes between the crust and the ocean^2^,^3^, and supports a vast and diverse crustal biosphere^4^,^5^. Basement outcrops allow massive hydrothermal flows to bypass marine sediments that generally have much lower permeability than the underlying volcanic rocks^6^,^7^,^8^,^9^. Although bare volcanic rock is common close to seafloor spreading centres, where the crust is young, widely spaced rock outcrops provide the primary pathways for hydrothermal exchange of fluid, heat and solutes between crust and the ocean on older and more heavily sedimented ridge flanks^8^,^10^,^11^,^12^. Flow between rock outcrops, which can be separated laterally by tens of kilometres, is driven by a hydrothermal siphon, where the primary impelling force is generated by the difference in density between recharging (cool) and discharging (warm) columns of crustal fluid^8^,^13^,^14^. However, factors controlling flow sustainability, rate and direction in these hydrothermal siphon systems have not previously been identified or explained.

Computer simulations of ridge–flank hydrothermal siphons can be used to determine the physical parameters that allow these systems to function, and how system properties influence fluid and heat transport. In comparison with earlier one and two-dimensional (2D) models of similar systems^8^,^12^,^14^, 3D simulations provide a more accurate representation of crustal and outcrop geometries, and lateral heat extraction adjacent to 3D fluid flow paths. In comparison with steady-state models, transient simulations include more realistic flow behaviours such as mixed convection and simultaneous axisymmetric and asymmetric flow around outcrops.

Here we present the first transient, 3D simulations of outcrop-to-outcrop hydrothermal siphons on the seafloor, and explore the parameter space under which a siphon is sustained. The outcrop geometry and the range of sediment and basement properties simulated are guided by conditions observed 100 km east of the Juan de Fuca Ridge^15^, northeastern Pacific Ocean, where thermal, geochemical and hydrogeological field observations show that a hydrothermal siphon is presently active^8^,^16^. Through these models, we identify key controls on system behaviour (outcrop size and permeability), and provide a mechanistic explanation as to why some outcrop-to-outcrop systems sustain hydrothermal siphons, whereas others do not. Simulations indicate that, for the geometry and range of properties tested, a significant contrast in outcrop properties is required for a hydrothermal siphon to be sustained, and that discharge is favoured through the outcrop that is more restrictive to flow. This helps to explain field observations indicating that small outcrops tend to be sites of hydrothermal discharge^8^,^11^,^17^, and suggests that small outcrops may play an especially important role in extracting lithospheric heat from the oceanic crust.

## Results

### Simulation configuration and dynamics

**Figure 1:**
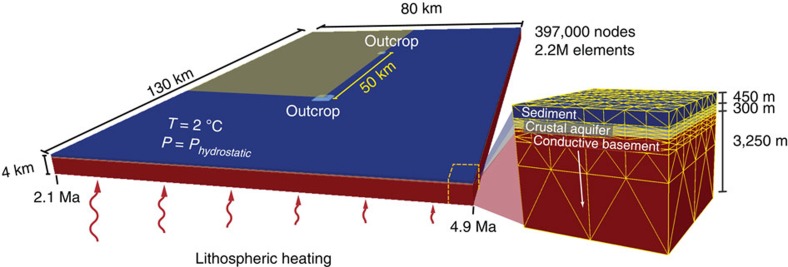
Geometry and configuration of 3D domains. Domains represent a section of upper ocean crust, oriented with the long-axis parallel to the spreading ridge, consistent with conditions at a field site on the eastern flank of the Juan de Fuca Ridge^8^,^14^,^16^. A conductive volcanic rock section (red, lower permeability) is overlain by a crustal aquifer (orange, higher permeability) and marine sediments (blue, lower permeability) and two volcanic rock outcrops penetrate through the sediment (light blue). Heat is applied to the base, following a lithospheric cooling trend (*1*). The sides and base are no-fluid flow boundaries, and the top is free flow (fluid and heat) with pressure varying as a function of seafloor depth. System properties are summarized in the Table 2. Simulation results within the volume delineated with the yellow rectangle are shown in Fig. 2.

Simulation domains are 130 km long, 80 km wide and 4 km thick, with no-flow side boundaries, lithospheric heating from below (varying with position according to crustal age), constant temperature at the top (seafloor) and seafloor pressure varying with water depth (Fig. 1). Two volcanic rock outcrops are separated by 50 km, penetrating upwards from a flat crustal aquifer and extending 65–500 m above an otherwise-continuous sediment layer. Most simulations are started with a pre-existing hydrothermal siphon running between the two outcrops, to distinguish the investigation of siphon sustainability from issues associated with initial siphon formation, although similar behaviours are observed in simulations that started from a hydrostatic initial condition (Supplementary Fig. 1). Each simulation is run until a dynamic steady state is achieved, wherein transient behaviours persist (for example, mixed convection, unstable secondary convection and local circulation), and recharge and discharge rates through outcrops stabilize to±0.1% per ky of simulation time.

**Figure 2:**
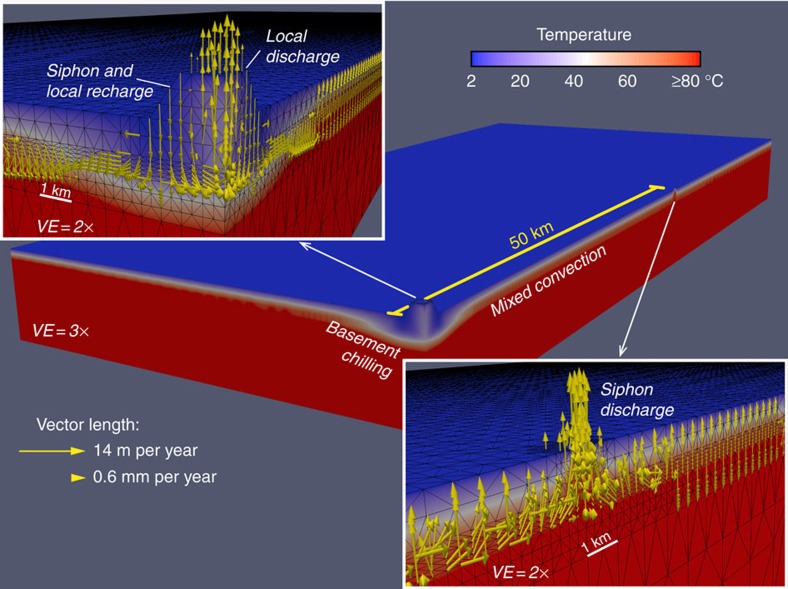
Simulation results at dynamic steady state. This simulation, showing one quarter of the domain illustrated in Fig. 1, has one large outcrop and one small outcrop (characteristics defined in Table 1). Domain colours show domain temperatures, including influence of rolling/mixed convection in basement aquifer and thermal influence of recharging/discharging outcrops. Inset diagrams show fluid flow vectors within and around outcrops (length indicates flow rate), with vectors plotted on a natural-log scale, the longest vector (exiting the top of the discharging outcrop) corresponding to a flow rate of ∼4.5 × 10^−7^ m s^−1^ (14 m per year). Fluid flow through the sediment is so slow that it would generate no detectable thermal or geochemical anomalies. Vertical exaggeration (VE) of main image is 3 × ; VE of inset images is 2 × .

Simulations are considered to satisfy key observational constraints when these regional flow characteristics are observed: a hydrothermal siphon operates between the two outcrops 50 km apart, discharging 5–20 kg s^−1^ of fluid and 1–3 MW of heat^18^,^19^,^20^, and does not result in regional heat-flux suppression at the seafloor^14^. For simulations that sustain a hydrothermal siphon, net lateral fluid transport in the upper crustal aquifer occurs from the recharge outcrop towards the discharge outcrop, although one or both outcrops may recharge and discharge fluids simultaneously (Fig. 2). There is a region of chilled aquifer surrounding the outcrop that recharges the hydrothermal siphon, extending laterally several kilometres from the edge of the volcanic edifice, whereas temperatures within the discharging outcrop are elevated by warm, rapidly ascending fluid (Fig. 2). Specific discharge (volume flow rate per cross-sectional area perpendicular to flow) within the outcrops and the aquifer between the outcrops is typically 1–20 m per year, with the highest flow rates seen within the discharge outcrop. These flows are driven by pressure differences in the crustal aquifer between the base of recharging and discharging outcrops of 20–100 kPa, consistent with the magnitude of differential pressures measured with subseafloor observatories on the eastern flank of the Juan de Fuca Ridge^21^. Simulations that are consistent with field observations also include a temperature difference between the bases of recharging and discharging fluid columns of ∼60 °C, mixed (unsteady and rolling) convection within the crustal aquifer between outcrops and insufficient fluid flow through seafloor sediments to cause measurable thermal or chemical perturbations (Fig. 2).

### Metrics of hydrothermal siphons

The mass rate of simulated siphon flow (*Q*_S_) is calculated by subtracting recharge from discharge at the discharge site. Thus, local recharge that contributes to discharge is not considered to be part of the hydrothermal siphon. The fraction of total outcrop discharge passing through the siphon (*F*_S_) is ≤0.75. Values of *F*_S_ tend to rise and fall with values of *Q*_S_, illustrating that when there is a greater rate of flow through the siphon, there also tends to be less simultaneous outcrop–local recharge and discharge. An upper crustal aquifer permeability (*k*_aq_) of ∼10^−12^ m^2^ is necessary to sustain the hydrothermal siphon and match typical flow characteristics at the field site (discharge of 5–20 kg s^−1^, no regional suppression of seafloor heat flux). Higher *k*_aq_ generally results in higher *Q*_*S*_ than observed, accompanied by excessive lowering of temperatures at the sediment–basement interface, and leading to excessive regional heat extraction, whereas lower *k*_aq_ results in lower *Q*_S_ or fails to sustain a hydrothermal siphon between outcrops.

**Figure 3:**
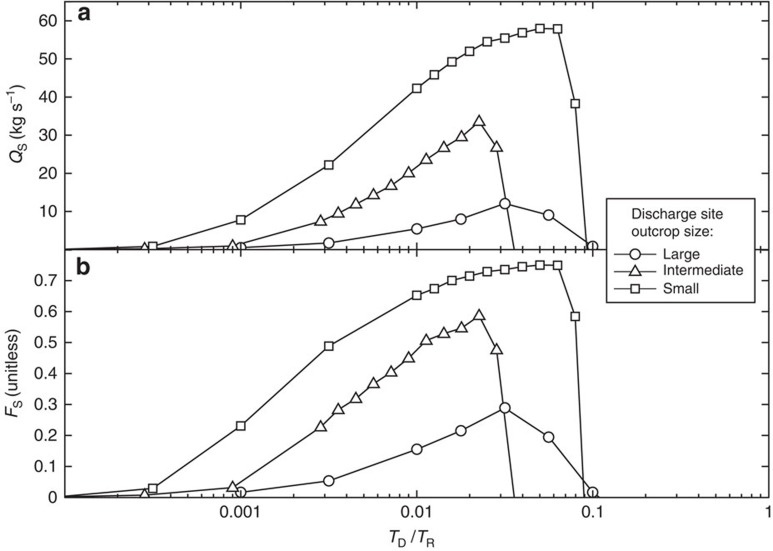
Outcrop-to-outcrop siphon behaviour for simulations having different outcrop sizes. (**a**) Siphon flow rate, *Q*_S_. (**b**) Siphon fraction, *F*_S_. Outcrop geometries defined in Table 1. Each point represents a simulation run to dynamic steady state. Crustal aquifer permeability is *k*_aq_=10^−12^ m^2^, and a large siphon–recharge outcrop (*A*=14.1 km^2^) has permeability *k*_oc_=10^−12^ m^2^, for all results shown. Permeabilities in the siphon–discharge outcrop differ for each simulation, as shown by the ratio of outcrop transmittance (*T*_D_/*T*_R_). Each geometry results in peaks for *F*_S_ and *Q*_S_ when 0.02<*T*_D_/*T*_R_<0.07, and siphons fail to sustain when *T*_D_/*T*_R_ >0.1.

**Figure 4:**
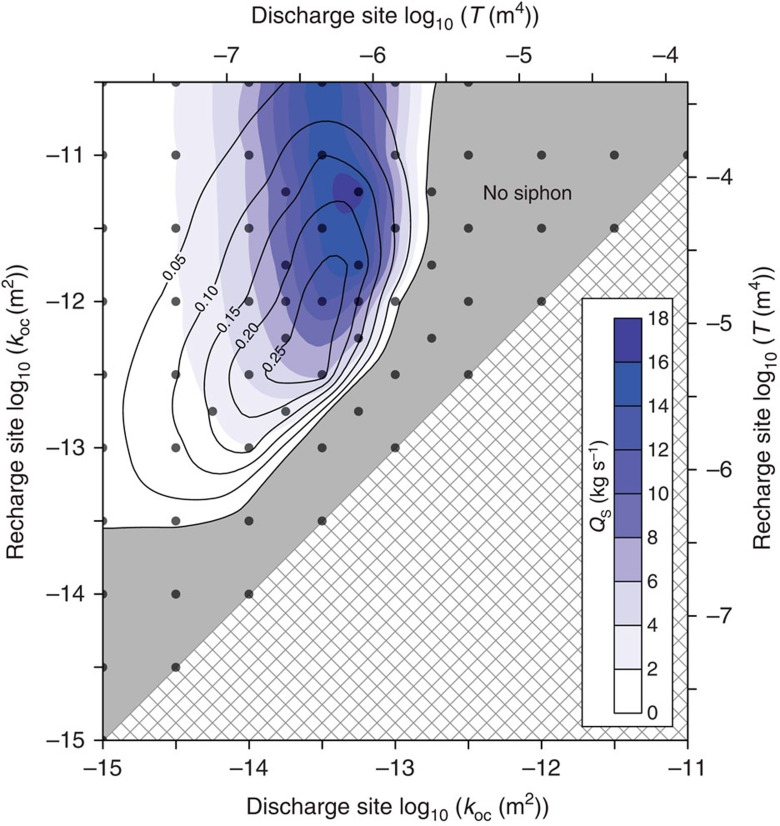
Outcrop-to-outcrop siphon behaviour for simulations with two large outcrops. Outcrop geometries are defined in Table 1. Bottom and left axes indicate outcrop permeability, whereas top and right axes show equivalent transmittance (permeability × outcrop area). Each circle represents results of a single simulation, run to dynamic steady state, delineating the permeability of recharge and discharge ends of the hydrothermal siphon. Colour contours illustrate *Q*_S_ (siphon flow rate), whereas solid contour lines and labels delineate *F*_S_ (siphon fraction). Note offset between peak values of *Q*_S_ and *F*_S_. Simulations within the grey zone did not sustain a hydrothermal siphon.

We define transmittance (*T*, m^4^) as the outcrop permeability times the area of outcrop exposure at the seafloor (*k* × *A*), a measure of the capacity of a rock outcrop to transmit fluid as part of a hydrothermal siphon. Two sets of simulations illustrate how outcrop properties affect siphon behaviour. In the first set, we modify *T* at the discharge site (variable *T*_D_) by changing both outcrop size and permeability (Table 1), while holding outcrop properties fixed at the recharge site (constant *T*_R_; Fig. 3). Hydrothermal siphons in these simulations transmit *Q*_S_≤60 kg s^−1^, with *Q*_S_ generally increasing with *T*_D_ until the siphon fails. In the second set of simulations, with two outcrops of equal size, we vary *T* by modifying outcrop permeability only. This second set of simulations generates similar behaviours to those seen in the first set, but lower siphon discharge rates (*Q*_S_≤18 kg s^−1^; Fig. 4). For both sets of simulations, hydrothermal siphons are sustained only when *T*_D_/*T*_R_ <0.1, with *Q*_S_ tending to be greatest at somewhat lower *T*_D_/*T*_R_ values (Fig. 4). In addition, every simulation that sustains a hydrothermal siphon does so with the lower-*T* outcrop becoming the primary site of siphon discharge, even when siphon flow is initiated in the opposite direction (alternative initial conditions discussed in Methods section).

## Discussion

These simulations demonstrate that, given sufficient permeability in the crustal aquifer, the variability of volcanic outcrop transmittance determines both (a) whether or not a hydrothermal siphon can be sustained and (b) the dominant siphon flow direction. A flow restriction at a ridge–flank discharge site (low *T*_D_) slows the overall rate of siphon transport, allowing the fluid to be warmed by lithospheric heat, which increases the impelling force for the siphon. At the same time, relatively high permeability in the crustal aquifer allows the pressure difference between recharging and discharging ends of the siphon to drive lateral flow with minimal energy loss. In cases where the difference in outcrop properties is smaller (*T*_D_/*T*_R_ >0.1), the pressure difference in the crust between the outcrops may be insufficient to overcome viscous losses in the intervening aquifer, rendering the siphon unsustainable. The abrupt transition in behaviour between systems that sustain siphons and those that do not (Fig. 3) indicates that the transmittance ratio exerts a fundamental control on siphon sustainability in systems of this kind. The transition occurs at *T*_D_/*T*_R_ ∼0.1 for the geometry and properties assigned in this study, but this transition will likely be different for systems having alternative outcrop spacing, aquifer thickness and/or permeability.

The finding that hydrothermal siphons tend to discharge through outcrops with lower *T* is consistent with field observations, suggesting that discharge is favoured through smaller outcrops^8^,^11^,^17^. In addition, it has been proposed that higher temperature (‘black smoker') hydrothermal vents on mid-ocean ridges tend to discharge at sites where there is a flow restriction^22^,^23^,^24^,^25^. Although mid-ocean ridge hydrothermal systems include many characteristics that are not found on volcanically inactive ridge flanks (for example, phase separation of flowing fluids, development of a cracking front and faster rates of reaction), the consistency of this trend could indicate a fundamental behaviour of subseafloor hydrogeological systems driven by lithospheric heat.

Although outcrop transmittance comprises the primary control on siphon behaviour in our simulations, outcrop size has an additional influence. Simulations having smaller outcrops as discharge sites yield higher *Q*_S_ and *F*_S_ than those with larger outcrops having equivalent *T*_D_ (Fig. 3). This may occur because, given a particular flow rate (limited mainly by system geometry, aquifer permeability and available heating from below), higher temperatures in ascending crustal fluids are thermodynamically easier to maintain in small outcrops than in large outcrops. A warmer column of discharging fluid creates a larger difference in fluid pressure between the base of recharging and discharging outcrops, generating larger lateral driving forces and flow rates within the underlying crust. Small outcrops also tend to be dominated by the thermal influence of one direction of fluid flow, as less space is available for flow paths to develop in both directions. Thus, once a small outcrop is established as a discharge site, local recharge (and associated crustal cooling) is inhibited, thereby boosting *F*_S_. These results suggest that outcrops smaller than ∼2 km in diameter, which are thought to be abundant globally but are generally undetectable with satellite gravimetric data^26^, may have a disproportionate influence on lithospheric heat extraction. This may explain why so few sites of ridge–flank hydrothermal discharge, a global process responsible for 25% of Earth's geothermal heat loss, have been identified to date: the vast majority of sites where this process occurs remain unmapped and unexplored.

In simulations that sustain a hydrothermal siphon through two larger outcrops (differences in *T* result entirely from differences in *k*), *F*_S_ is generally low enough to allow significant outcrop–local circulation (Fig. 4). Both outcrops generate local recharge and discharge in these simulations, even in those sustaining a hydrothermal siphon. Simultaneous recharge and discharge through large outcrops that are thought to be sites of hydrothermal siphon recharge has been observed at field sites^11^,^14^,^27^. That the hydrothermal siphon fails when *T*_D_/*T*_R_ >0.1 demonstrates the additional possibility that local (single-outcrop) hydrothermal circulation systems can develop within proximal outcrops between which there is no siphon flow, even if there is a permeable aquifer connecting them.

The minimum aquifer permeability required to sustain an outcrop-to-outcrop hydrothermal siphon in this study, *k*_aq_*=*10^−12^ m^2^, is well represented by the global data set of *in situ* permeability measurements in the upper ocean crust (Supplementary Fig. 2). This permeability value is at the lower end of values estimated with 1D analytical calculations^7^,^8^, and lower than inferred from 2D simulations based on an equivalent geometry^14^. Three-dimensional numerical simulations may result in more fluid flow and heat extraction than do 1D and 2D simulations with equivalent permeability because the 3D simulations result in advective heat extraction focused within a comparatively small area. This allows recharging and discharging fluid columns in the crust to be relatively isothermal, maximizing the driving force for siphon flow. In contrast, 2D simulations treat volcanic outcrops as ‘ridges' that extend to infinity in and out of the plane of the simulation, so a smaller fraction of crustal heat is advected per area of 2D outcrop, and higher aquifer *k*_aq_ is required for the siphon to be sustained.

The simulations presented in this study were developed for an end member of ridge–flank hydrothermal systems, in which there is a relatively low flow rate (tens of kg s^−1^) that mines little lithospheric heat, based on a field site where there is considerable understanding of system geometry, crustal properties and flow fluid rates^8^,^14^,^15^,^18^,^19^. The hydrothermal siphon at this field site likely operated much more vigorously in the past when sediment cover was thinner and less extensive, and there was a larger network of exposed basement outcrops^28^. On a global basis, outcrop-to-outcrop circulation systems are generally more efficient at extracting lithospheric heat than is the system operating presently at this field site^1^,^6^,^7^,^12^. Based on trends from simulations presented herein (Figs 3 and 4), higher fluid flow rates between outcrops cannot be generated by larger differences between *T*_R_ and *T*_D_ alone (holding other parameters with ranges simulated). Instead, achieving greater basement cooling and a larger reduction in seafloor heat flux likely requires higher aquifer permeability and/or an outcrop geometry that allows faster fluid flow rates through the upper crust. We hypothesize that one or both of these conditions will help to explain the global heat-flux anomaly on ridge flanks, and may account for even more extreme cases of highly efficient heat extraction from these systems^6^,^11^,^15^.

## Methods

### Computational methods

The numerical model used in this study, Finite Element Heat and Mass (FEHM), uses a finite volume representation of the physical domain^29^. FEHM is node-centred and was run with a Delaunay mesh of tetrahedral elements, incorporating a finite volume approach to representing properties and flows between nodes. Darcy's law governs (laminar) fluid flow in these simulations, and flow rates calculated in the present study are consistent with this representation. FEHM is fully coupled and transient, with flow potential and fluid properties (and thus the vector components defining the 3D flow field) being updated with each time step. We applied FEHM with a solver that is fully implicit with upstream weighting.

All simulations were run for ≥10^5^ years of simulation time (1,000–2,000 time steps), sufficient to reach a dynamic steady state such that recharge and discharge rates from outcrops stabilized to ±0.1% per ky of simulation time, requiring runtimes of 1–10 days on a desktop (Linux) workstation. Whole-grid mass and energy conservation were confirmed for simulations at dynamic steady state, finding the apparent ‘imbalance' between input and output to be <3% at late times. These apparent imbalances arise mainly because of very low rates of fluid transport (at rates too small to be detected in the field, <0.1 mm per year) across a large seafloor area. These very slow flows comprise a percentage of flow through outcrops only because outcrop flows are relatively small for the field setting used as the basis for simulations. Apparent imbalances also occur because flows through the whole crustal system (>4 × 10^4^ km^3^) continue to be transient and oscillatory, with water moving into and out of storage throughout the simulations. Internal mass balance errors, calculated by FEHM during solution iteration, are «1%.

### Grid development and resolution

Grid geometries for simulations shown in this study comprise ∼4 × 10^5^ nodes and ∼2.2 × 10^6^ elements. Initial simulations were performed using coarser grids, which yielded somewhat different local convection patterns, but siphon flow and sustainability (the focus of this study) were otherwise robust to differences in grid resolution. Grid resolution is highest within the aquifer and outcrops, with typical node spacing of 50–225 m, consistent with grid spacing used in earlier, 2D models of ridge–flank hydrothermal systems^14^,^30^,^31^,^32^,^33^. Cells are coarser within the sediment (200–500 m node spacing) and the low-permeability basalt layer underlying the aquifer (150–2,000 m spacing), both of which experience no measurable fluid flow based on thermal and/or geochemical measurements (≤0.1 mm per year). Areas of the domain located ≥10 km horizontally from a volcanic rock outcrops also have larger node spacing to improve computational efficiency. The relatively thick section of low-permeability volcanic rock below the crustal aquifer allows the conductive redistribution of lithospheric heat rising from depth, which is important for capturing the full coupling between hydrothermal circulation, patterns of advective heat extraction and conductive seafloor heat flux.

Outcrop geometries are patterned after those observed on 3.5 My old seafloor on the eastern flank of the Juan de Fuca Ridge^8^,^15^ are and simulated as ziggurats (flat-topped pyramids). The smallest outcrops represented in our simulations have a geometry similar to that of Baby Bare outcrop^18^,^19^, which rises 65 m above the surrounding sediments but is the tip of a much larger volcanic edifice that is mostly buried by regionally thick and continuous turbidites and hemipelagic mud. The largest outcrops in our simulations have a geometry similar to Grizzly Bare outcrop^14^,^27^, rising ∼500 m above the surrounding seafloor. The ratio of outcrop areas for the large and small outcrops in our simulations is ∼100, and the medium-sized outcrops simulated in this study have an area and elevation that is intermediate to that of the large and small outcrops (Table 2).

### Initial conditions

Most simulations were started with the initial temperature and pressure conditions of an active outcrop-to-outcrop hydrothermal siphon. Generating this state requires a series of steps, starting with a conduction only (no-fluid flow) simulation that yields a thermal state consistent with the simulated geometry and physical properties. We use these results to calculate hydrostatic pressures consistent with conductive thermal conditions at each node as a function of depth, including variations in fluid density with temperature and pressure (‘conductive–hydrostatic'). These initial conditions are used to start a fully coupled simulation that can spontaneously form an outcrop-to-outcrop hydrothermal siphon, given appropriate aquifer and outcrop permeabilities. We use the fluid and formation pressure and temperature conditions that result from this flowing hydrothermal siphon as a consistent starting condition for all subsequent simulations of the same crustal geometry.

We also performed a suite of simulations in which the initial condition was ‘conductive–hydrostatic,' rather than being based on an active outcrop-to-outcrop siphon. In almost every case, the simulated behaviours and *Q*_S_, at dynamic steady state when starting from ‘conductive–hydrostatic,' were identical to those based on starting with an active siphon (Supplementary Fig. 1). The exception was for a single simulation with different-sized outcrops and *T*_D_/*T*_R_ ∼0.1, in which an initial siphon can self-sustain under conditions where it would not develop spontaneously from ‘conductive–hydrostatic.' We also ran subset of the simulations presented in Figs 3 and 4 beginning from an active siphon that flows in the opposite direction. In all of these cases, the siphon either failed or spontaneously switched direction to yield identical results at dynamic steady state to those started from ‘conductive–hydrostatic.' We focus in this study on sustaining a hydrothermal siphon, rather than developing it from ‘conductive–hydrostatic,' to avoid convolving the influence of system properties and initial conditions on siphon behaviour. Real outcrop-to-outcrop hydrothermal siphons develop through a complex history of volcanism, lithospheric cooling, sedimentation, consolidation, lithification, alteration and tectonic processes, all of which are highly variable and details of which are poorly known at individual field sites.

### Physical properties

The physical properties assigned to parts of each grid are summarized in Table 2. Individual model cells are intended to comprise representative elemental volumes, in which bulk properties apply to solid material and pore space surrounding a single node. We use homogenous and isotropic bulk properties for the volcanic (basaltic) crust, with a single value assigned to each section (the crustal aquifer, the low-permeability crustal layer beneath and two outcrop regions).

Sediment porosity, thermal conductivity and permeability vary with depth to account for compaction. We used data from the field area to create representative depth-dependent functions for each property. Each function was discretized and values were assigned to nodes so that the cumulative effects of the sediment layer were accurately represented. Although there is some uncertainty in the estimates of bulk sediment properties, we find that the results of this study are robust to realistic deviations in the hydrological and thermal properties assigned.

The values chosen for basement permeability and thickness of the crustal aquifer were based on consideration of the global borehole data set of *in situ* permeability measurements (Supplementary Fig. 2), and simulations that resulted in either no-sustained hydrothermal siphon (*k*_aq_ ≤10^−13^ m^2^) or siphon flow rates and cooling within the upper crustal aquifer exceeding observations at the field site (*k*_aq_≥ 10^−11^ m^2^). *In situ* bulk permeability determinations made in basaltic ocean crust, using an inflatable packer or a temperature log in an unsealed hole, indicate permeability in the upper 300 m below the sediment–basement interface having a range of 10^−14^ to 10^−10^ m^2^ (Supplementary Fig. 2). There are few crustal permeability measurements that extend below the upper 300 m of basement, but these suggest somewhat lower values. Simulation results shown in the present study are based on crustal aquifer thickness of 300 m, which is consistent with the global permeability data set. Additional simulations show that results are comparable for thicker or thinner crustal aquifers, if the *k*_aq_ is proportionately adjusted such that the product of aquifer thickness and permeability remains the same.

## Additional information

**How to cite this article:** Winslow, D.M. and Fisher, A.T. Sustainability and dynamics of outcrop-to-outcrop hydrothermal circulation. *Nat. Commun.* 6:7567 doi: 10.1038/ncomms8567 (2015).

## Supplementary Material

Supplementary Information Supplementary Figures 1-2 and Supplementary References.

## Figures and Tables

**Table 1:** Volcanic rock outcrop characteristics used in coupled-flow simulations.

	Exposure area* ***A*** **(km**^**2**^)	**Height**† **(m)**	**Top width**‡ **(km)**	**Base width**‡ **(km)**
Small	0.141	65	0.25	1.0
Intermediate	1.27	200	0.50	2.0
Large	14.1	500	2.0	4.5

^*^Map-view cross-sectional area of outcrop exposed at the seafloor.

^†^Outcrop height above the seafloor.

^‡^Top and base widths are measured side to side, with base width measured at the sediment–basement interface. Outcrop edifices are simulated as ziggurats (flat-topped pyramids).

**Table 2:** Formation properties used in coupled-flow simulations.

	**Porosity,** ***n*****(unitless)**	**Thermal conductivity,** ***λ*****(W m**^**−1**^** K**^**−1**^)	**Permeability,** ***k*****(m**^**2**^)
Sediment*	0.39–0.52	1.36–1.51	1.1 × 10^−17^ to 2.2 × 10^−17^
Outcrop†‡	0.1	1.82	1 × 10^−15^ to 3.2 × 10^−11^
Aquifer†	0.1	1.82	10^−12^
Deep crust†	0.05	1.93	10^−18^

^*^Values vary with depth and are consistent through all simulations. The relatively narrow range of sediment permeabilities applies to the full-sediment column, representing a range at a smaller scale of several orders of magnitude.

^†^Values assigned homogeneously throughout each region.

^‡^Each outcrop is assigned a single value in a given simulation. Range refers to values assigned across all simulations.
