# Supplementary material for: Sustainability and dynamics of outcrop-to-outcrop hydrothermal circulation
Source: Nat Commun. 2015 Jun 26;6:7567. doi: 10.1038/ncomms8567 (PMC4491839; doi:10.1038/ncomms8567)
Supplement: Supplementary Information — Supplementary Figures 1-2 and Supplementary References. [file ncomms8567-s1.pdf]

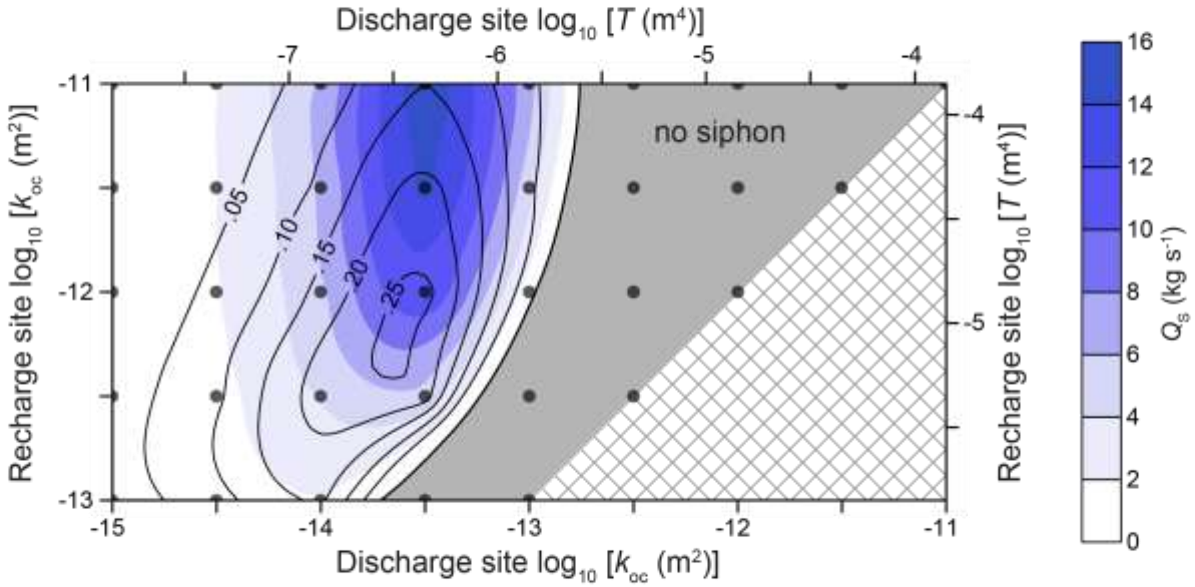

Supplementary Fig. 1. Outcrop-to-outcrop siphon behavior for simulations with two large outcrops. Simulations are as shown in Fig. 4, but started from a "conductive-hydrostatic" initial conditions rather than a running siphon. Each circle represents results of a single simulation, run to dynamic steady state, delineating the permeability of recharge and discharge ends of the hydrothermal siphon. Solid contour lines and labels delineate F_S; filled color contours illustrate Q_S.

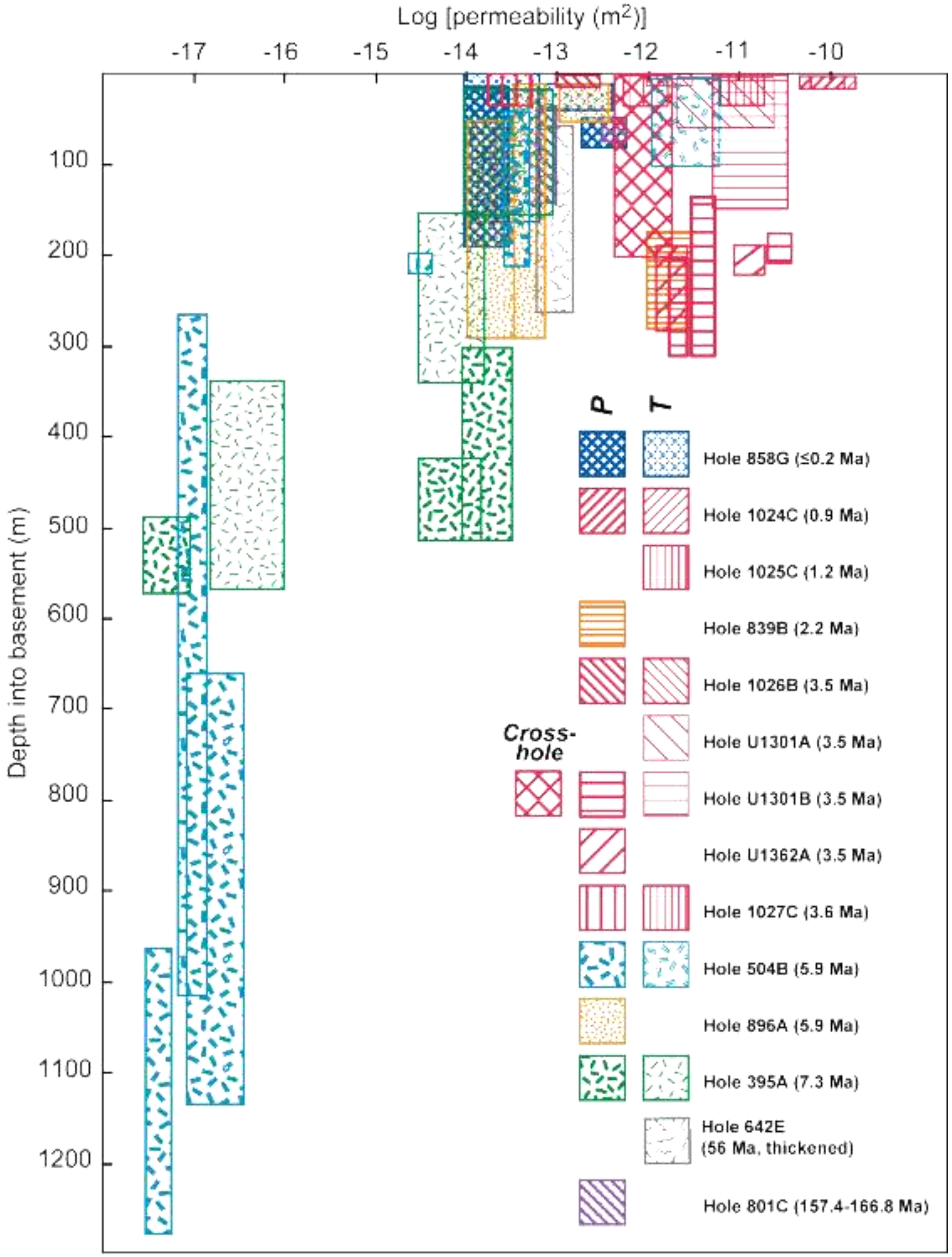

Supplementary Fig. 2. Compilation of borehole measurements of permeability in the basaltic (volcanic) ocean crust. Figure modified from ref (1). Data in this compilation are from packer experiments[2] (*P*), modeling of borehole thermal logs[3] (*T*), and a single cross-hole response experiment[4], as labeled.

Supplementary References

1. Fisher, A. T., Alt, J. C. & Bach, W. in *Earth and Life Processes Discovered from the Subseafloor Environment - A Decade of Science Achieved by the Integrated Ocean Drilling Program (IODP)* (eds. Stein, R., Blackman, D., Inagaki, F. & Larsen, H. C.) 507–551 (Elsevier, 2014).
2. Becker, K., Fisher, A. T. & Tsuji, T. New packer experiments and borehole logs in upper oceanic crust: Evidence for ridge-parallel consistency in crustal hydrogeological properties. *Geochem. Geophys. Geosystems* **14,** 2900–2915 (2013).
3. Winslow, D. M., Fisher, A. T. & Becker, K. Characterizing borehole fluid flow and formation permeability in the ocean crust using linked analytic models and Markov chain Monte Carlo analysis: Borehole Flow and Formation Permeability. *Geochem. Geophys. Geosystems* **14,** 3857–3874 (2013).
4. Fisher, A. T., Davis, E. E. & Becker, K. Borehole-to-borehole hydrologic response across 2.4 km in the upper oceanic crust: Implications for crustal-scale properties. *J. Geophys. Res.* **113,** (2008).
